# Corneal Transplant Infection due to *Alternaria alternata*: A Case Report

**DOI:** 10.1155/2013/589620

**Published:** 2013-03-14

**Authors:** Vasileios Konidaris, Andreana Mersinoglou, Timoleon-Achilleas Vyzantiadis, Domniki Papadopoulou, Kostas G. Boboridis, Panagiotis Ekonomidis

**Affiliations:** ^1^Ophthalmology Department, AHEPA University Hospital, Aristotle University of Thessaloniki, St. Kyriakidis 1 Street, 54636 Thessaloniki, Greece; ^2^Department of Microbiology, Medical School, Aristotle University of Thessaloniki, 54124 Thessaloniki, Greece

## Abstract

*Purpose*. To report a case of *Alternaria alternata* keratitis in a patient with a corneal transplant in her right eye due to bullous pseudophakic keratopathy. *Methods*. A 66-year-old female underwent a full-thickness keratoplasty in her right eye due to bullous pseudophakic keratopathy. Three weeks after keratoplasty, epithelial edema and a stromal opacity with an infiltrate and development of peripheral corneal opacities appeared. The diagnosis of *Alternaria alternata* keratitis was made. *Results*. The patient underwent a second keratoplasty, due to the corneal melting as a result of the fungal infection. She was also given combined antifungal treatment locally and systematically. *Conclusion*. Corneal transplantation alone would not have been sufficient to keep the fungus in the anterior portion of the eye. Combined antifungal treatment, locally and systematically, was important in attempting to prevent the further spread of the fungus to the interior of the eye. To our knowledge, the case presented here is only the second one in the literature concerning a keratomycosis due to *Alternaria alternata* corneal transplant infection.

## 1. Introduction

*Alternaria alternata *is a saprophytic dematiaceous mould, found mostly in soil and decomposing vegetation [1]. Among *Alternaria* species, it is the most commonly isolated from human infections [2]. It is a known agent of ocular infections and has been reported to cause both keratitis and endophthalmitis [3, 4]. *Alternaria *keratitis was first reported in 1975; the patient required penetrating keratoplasty, and the graft eventually failed [5]. The most common predisposing factor appears to be the trauma [6]. 

## 2. Materials and Methods

A 66-year-old female presented due to pain and progressively decreased visual acuity in the right eye that had begun about 6 months prior. Both her eyes had undergone cataract surgery 3 years ago. Her visual acuity was counting fingers at a distance of 50 cm from the right eye, while that of the left eye was 0.7 measured in Snellen chart. Bullous pseudophakic keratopathy was found in the right eye. Corneal opacity was observed in all layers of the cornea, and epithelial bullas were found in the center of the right cornea. Vitreous and fundus could not be examined in the right eye due to the media opacities, while in the left eye were normal.

An uncomplicated penetrating keratoplasty was performed in the right eye. During the operation, povidone-iodine was used as a prophylaxis measure on the ocular surface, while 1 mL cefazolin (500 mg/2 mL) was injected subconjunctively after the surgery. Topical treatment was eye drops of dexamethasone every two hours, eye drops of ofloxacin every 6 hours, artificial tears hourly, and lubricant eye gel every 6 hours. The patient was also treated with per os methylprednisolone, 50 mg once per day, in tapering doses.

## 3. Results

**Figure 1:**
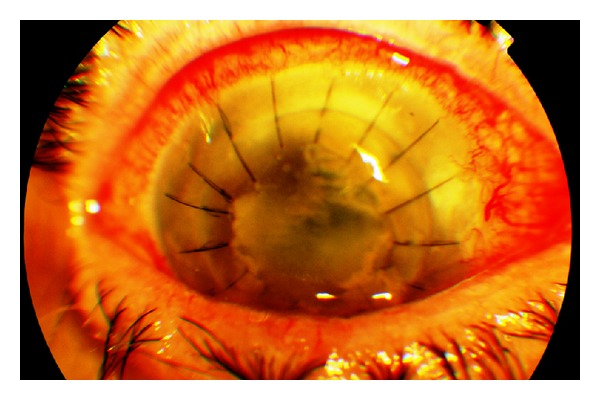
Epithelial edema and stromal opacity with an infiltrate and development of peripheral opacities three weeks after keratoplasty in the right eye.

Visual acuity was maintained at 0.1 during the first three weeks after surgery. Ocular pain recurred after that period, and visual acuity deteriorated to the level of hand motion perception. Trauma or systemic diseases were excluded. Slit-lamp biomicroscopy showed recurrence of epithelial oedema and stromal opacity with an infiltrate and development of peripheral opacities (satellite lesions) (Figure 1). Ocular ultrasound was performed, showing normal images of the vitreous.

Due to the clinical appearance, a fungal infection was suspected, and, consequently, corticosteroid treatment was discontinued. Drops of 1% voriconazole hourly were added. 

**Figure 2:**
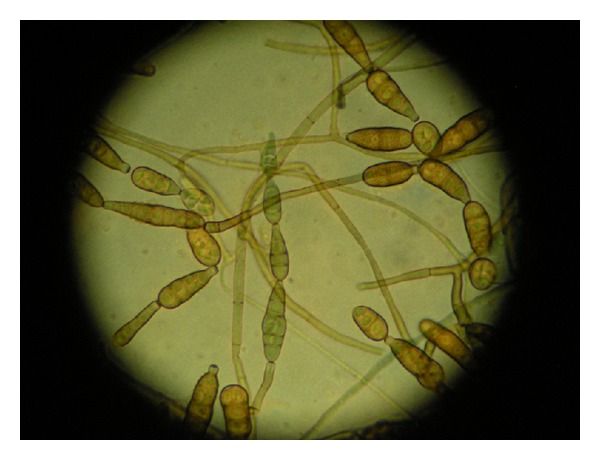
Tape mount with lactophenol-cotton blue. Chains of conidia with multiple septa (×400).

Corneal scrapings were collected from the margin of the abscess and from the bed of the corneal ulcer and sent for mycological examination. The specimen was inoculated onto Sabouraud dextrose agar with chloramphenicol (SDCA) as well as malt extract agar (MEA) and incubated at 30°C. Unfortunately, the specimen quantity was inadequate for direct microscopy observation. In both media, a moderate growth of a dark-colored mould was observed, filling the plates completely in three to four days. A subculture was performed onto potato dextrose agar (PDA) in order to further promote the fungal sporulation. The colonial appearance was that of a floccose mould with a whitish, at first, color, later becoming grey to olivaceous on PDA but more brownish on SDCA and MEA. The reverse was olivaceous for PDA and darker green-brown for SDCA and MEA. Microscopy revealed long chains of abundant club-shaped conidia with multiple transverse, longitudinal, and oblique septa. Many conidia were presented with an apical beak at their distal end. The conidiophores were mostly unbranched without secondary conidiophores (Figure 2). The size of the fungal structures was measured with the help of an eyepiece graticule. According to the above characteristics and with the use of identification keys, the mould was identified as *Alternaria alternata*. 

Antifungal susceptibility testing was performed by Etest strips (bioMérieux S.A., Marcy-l'Etoile, France) on RPMI-1640 MOPS agar plates, which revealed a minimum inhibitory concentration (MIC) of 0.047 *μ*g/mL for amphotericin B, 16 *μ*g/mL for flucytosine, 0.023 *μ*g/mL for itraconazole, 0.125 *μ*g/mL for voriconazole, 0.064 *μ*g/mL for posaconazole, and 0.125 *μ*g/mL for caspofungin. According to the results the strain was regarded susceptible to amphotericin B, itraconazole, voriconazole, posaconazole and caspofungin, and resistant to flucytosine.

**Figure 3:**
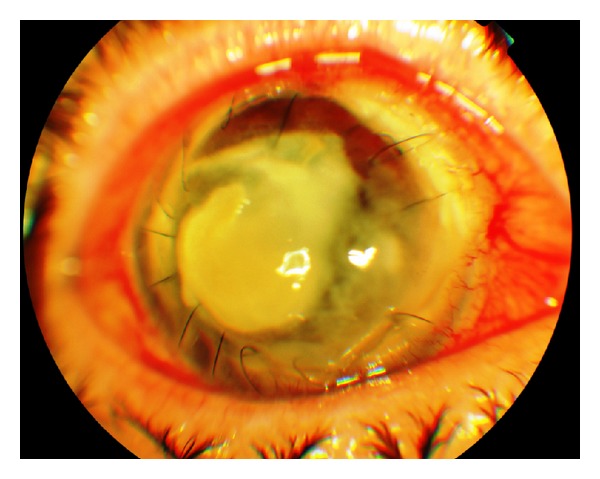
Central ulceration and abscess with indistinct margins, involving the central corneal zone.

Meanwhile, a growing in extension and depth central ulceration and an abscess with indistinct margins, involving the central corneal zone, were presented. As the laboratory results were not still confirmatory, the treatment was based on the abrupt onset of symptoms described by the patient as well as the clinical aspects. Despite the therapeutic regimen described previously, there was not any improvement of the cornea, and, moreover, it was actually enlarged (Figure 3). 

After the confirmation of a fungal involvement and the full morphological identification of the pathogen, eye drops of 0.15% *amphotericin* B/6 h and iv voriconazole at a dose of 150 mg every 12 h were added to the treatment. However, the infiltrate was already expanding, and the cornea started to melt. The patient underwent a second penetrating keratoplasty. It was performed with removal of a 1 mm larger disc area in order to encompass the previous graft and the host cornea affected by the ulceration and thinning.

Despite corneal edema, visual acuity was maintained at 0.08 during the first six months after the second surgery. During that period, the treatment was consisting of eye drops of 0.15% amphotericin B/6 h and voriconazole hourly as well as i.v. voriconazole (150 mg every 12 h).

## 4. Discussion

It has been demonstrated that the use of topical corticosteroid may predispose to the onset and the worsening of the fungal infection of the eye. Such incidents do not result in ulcer unless there is a breach in the corneal epithelium, delay in healing that provides a “window of opportunity” [7], inoculation of the microorganism, and development of subsequent ulceration. There has been reported that 36% of *Alternaria *keratitis cases resulted in either therapeutic penetrating or lamellar keratoplasty, despite the use of antifungal agents [8]. The therapeutical target in our patient case was to confine the infection within the graft, to stabilize the graft, and in a second time to replace it. Although the patient had to undergo a second keratoplasty within one month, the antifungal treatment appeared to have controlled the spread of *Alternaria *and, thus, minimized the risk of a permanent eye loss. 

The role of timely and aggressive medical intervention is of utmost importance to preserve the vision in the case of fungal keratitis. In this case, the fungal corneal infection was treated with a combination of amphotericin B and voriconazole. A study by Thiel et al. [9] demonstrated that orally administered voriconazole reaches therapeutic aqueous and vitreous levels in the noninflamed human eye. The clinical efficacy of voriconazole in keratitis caused by *Alternaria* has recently been reported [2]. To our knowledge, the case presented here is only the second one in the literature concerning a keratomycosis due to *Alternaria alternata* corneal transplant infection. In the first reported case by Ando and Takatori [10], a 53-year-old woman was found to have an ulcer on her successfully transplanted corneal graft which was attributed to an *Alternaria alternata* infection. In our case, diagnosis was complicated due to differences in clinical presentation after the penetrating keratoplasty but eventually the diagnosis of a fungal infection was made after the laboratory results revealed the existence of the fungus.

Transplantation of the cornea alone would not have been sufficient to keep the fungus in the anterior portion of the eye, because the periphery of the cornea was already infected. The preemptive combined antifungal treatment, locally and systematically, was important in attempting to prevent the further spread of the fungus to the interior of the eye.
